# A logNII-based model for predicting pathological complete response following neoadjuvant therapy in esophageal squamous cell carcinoma: a retrospective single-center study

**DOI:** 10.1186/s12957-026-04416-1

**Published:** 2026-05-30

**Authors:** Min Zhang, Jiahao Huang, Chunyan Wang, Bin Yang, Tao Lu

**Affiliations:** 1 Cancer Hospital Affiliated to Shanxi Medical University, Taiyuan, Shanxi Province China; 2 Department of Thoracic Surgery, Shanxi Province Cancer Hospital/ Shanxi Hospital Affiliated to Cancer Hospital, Chinese Academy of Medical Sciences/ Cancer Hospital Affiliated to Shanxi Medical University, Taiyuan, China; 3 Department of Clinical Laboratory, Shanxi Province Cancer Hospital/ Shanxi Hospital Affiliated to Cancer Hospital, Chinese Academy of Medical Sciences/Cancer Hospital Affiliated to Shanxi Medical University, Taiyuan, China

**Keywords:** Esophageal Cancer, Pathological complete response, Neoadjuvant immunochemotherapy, Nomogram model

## Abstract

**Objective:**

This study aimed to develop and validate a predictive model for pathological complete response (pCR) following neoadjuvant therapy in esophageal squamous cell carcinoma (ESCC), focusing on Nutrition-Inflammation Index (NII) and its potential nonlinear relationship with pCR.

**Methods:**

A single-center retrospective cohort of 363 ESCC patients receiving neoadjuvant therapy followed by esophagectomy was analyzed. We employed restricted cubic splines within multivariable logistic regression to characterize the relationship between log-transformed Nutrition-Inflammation Index (logNII) and pCR. The model’s performance was rigorously assessed by its discriminative ability (Area Under the Curve, AUC), calibration, and clinical utility using bootstrap validation and decision curve analysis (DCA).

**Results:**

The logNII demonstrated a significant, independent, and nonlinear association with pCR after adjusting for key clinical covariates. The final predictive model, which incorporated logNII and clinical variables, achieved an AUC of 0.816 (95% CI: 0.770–0.863). DCA confirmed the model provided significant net clinical benefit across a wide range of threshold probabilities, highlighting its potential for clinical decision-making.

**Conclusion:**

We established logNII as a robust, independent, and nonlinear predictor of pCR in ESCC. The developed model demonstrates excellent predictive performance and clinical utility, offering a valuable tool for personalizing treatment strategies in the neoadjuvant setting.

## Highlights

A novel tissue-based marker (logNII) independently predicts pathological complete response (pCR) in esophageal cancer.

The relationship between logNII and pCR is nonlinear, with risk rising sharply at higher levels.

A model combining logNII with clinical factors shows excellent prediction accuracy (AUC = 0.816).

Decision curve analysis confirms the model adds significant clinical benefit for treatment decisions.

## Introduction

Esophageal squamous cell carcinoma (ESCC) presents a significant global health burden, with particularly high incidence in East Asia’s “esophageal cancer belt,” where it comprises nearly 90% of all esophageal malignancies [1, 2]. Current statistics indicate approximately 400,000 annual deaths worldwide, with five-year survival rates remaining disappointingly low at 15–25% for advanced-stage disease [1, 2]. For locally advanced ESCC, neoadjuvant therapy followed by esophagectomy represents the standard treatment paradigm [3–6] However, treatment response shows substantial heterogeneity, with only 25–40% of patients achieving pathological complete response (pCR)—a well-established predictor of superior long-term survival [7]. This variability underscores the urgent need for reliable predictive biomarkers to guide personalized treatment strategies [8–12].

The scientific community has increasingly focused on identifying predictive biomarkers for treatment response in ESCC [8–12]. Current research encompasses a broad spectrum of hematological, molecular, and pathological parameters. Beyond the conventional inflammatory markers including neutrophil-to-lymphocyte ratio (NLR), platelet-to-lymphocyte ratio (PLR), and lymphocyte-to-monocyte ratio (LMR), more complex indices such as the systemic immune-inflammation index (SII) and pan-immune-inflammation value (PIV) have emerged as potential predictors [8, 9, 12]. In parallel, emerging modalities such as circulating tumor DNA (ctDNA) dynamics and radiomic nomograms have been explored for preoperative response assessment [10, 11] However, despite this extensive investigation, most biomarkers demonstrate inconsistent performance across validation cohorts, with area under the curve values typically ranging from 0.60 to 0.75 in predictive models. This limited discriminative capacity, combined with susceptibility to non-malignant conditions such as concurrent infections, inflammatory disorders, nutritional deficiencies, and other comorbidities, significantly compromises their clinical utility. Furthermore, these circulating biomarkers fail to capture the complex spatial architecture and functional state of the local host immune and inflammatory milieu, which represents a critical determinant of therapeutic response. The tumor microenvironment comprises a sophisticated ecosystem where immune cells, stromal elements, and tumor cells engage in dynamic crosstalk that ultimately dictates treatment outcomes [13–17].

Compared with previously reported systemic inflammatory or nutritional markers, the Nutrition-Inflammation Index (NII) is a composite laboratory index derived from routine preoperative blood and biochemical parameters, including lymphocytes, neutrophils, albumin, and alanine aminotransferase (ALT). Lymphocyte count reflects host immune competence and adaptive anti-tumor response, whereas neutrophil count may indicate a pro-inflammatory and immunosuppressive state associated with unfavorable tumor behavior. Albumin serves as a marker of nutritional reserve and systemic inflammatory burden, both of which may influence treatment tolerance and response. ALT was included as an indicator of hepatic and metabolic status that may be relevant to systemic inflammatory balance and host fitness during neoadjuvant therapy. By integrating these routine laboratory parameters, NII may provide a broader assessment of host inflammatory, immune, nutritional, and metabolic status than any single biomarker alone. Importantly, NII is a blood-based composite index and does not directly measure the tumor microenvironment. This study therefore evaluated the predictive value of log-transformed NII (logNII) for pathological complete response in ESCC patients undergoing neoadjuvant therapy [13–17].

## Methods

### Patient selection

This single-center retrospective cohort study was approved by the Ethics Committee of Shanxi Province Cancer Hospital (Ethics Approval Number: KY2024130), and the research process adhered to the ethical principles of the Declaration of Helsinki. The study included 363 patients with esophageal squamous cell carcinoma (ESCC) who underwent radical esophagectomy at our hospital between January 2022 and October 2025, following treatment with either neoadjuvant immunochemotherapy (NICT) or neoadjuvant chemotherapy (NCT).

**Fig. 1:**
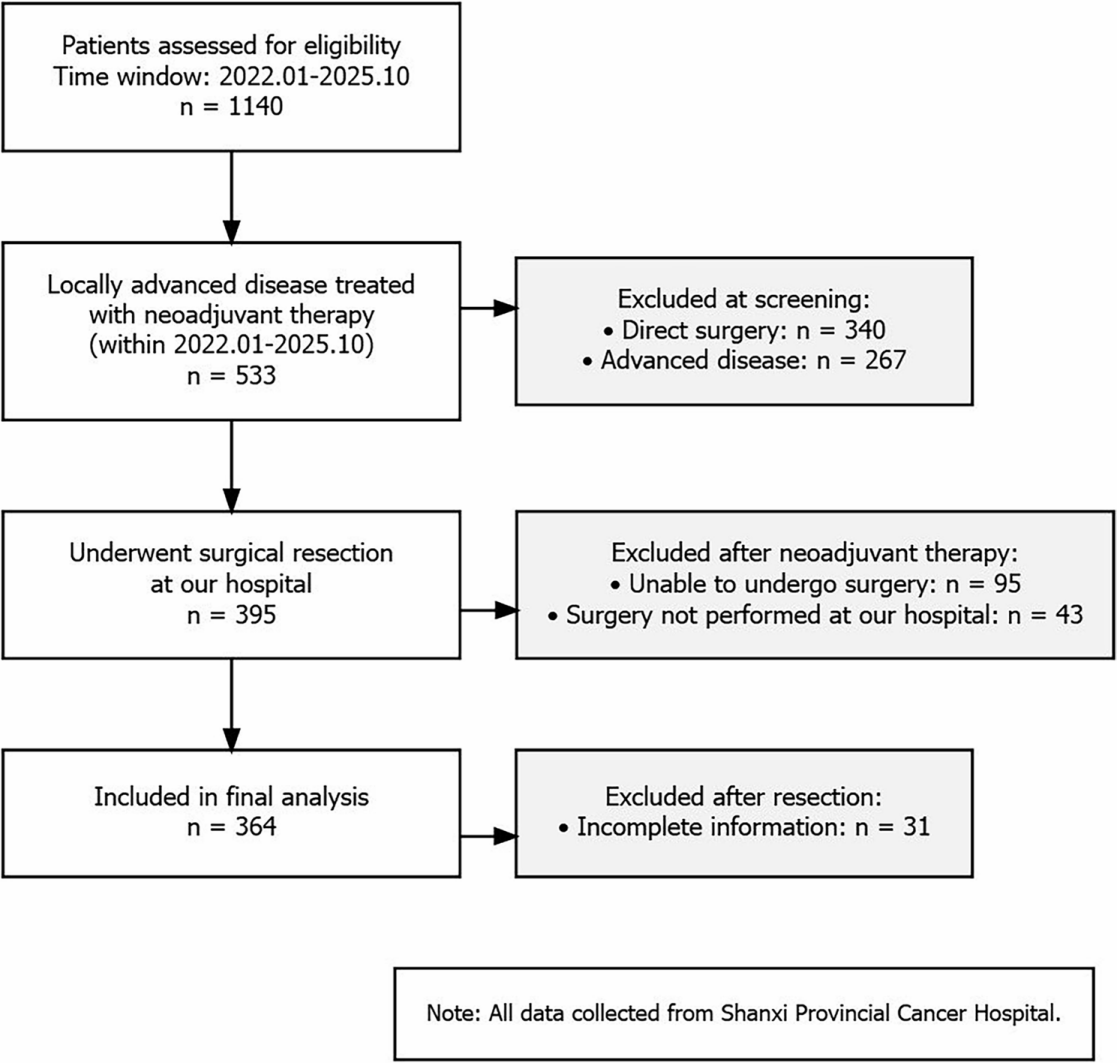
Flowchart of patient selection. The diagram illustrates the process of patient inclusion and exclusion for the study. Patients with locally advanced disease who received neoadjuvant therapy between January 2022 and October 2025 were assessed. Those who underwent surgical resection at our hospital and had complete clinical data were included in the final analysis. Abbreviations: n, number of patients

Inclusion criteria: (1) Pathological confirmation of ESCC by preoperative endoscopic biopsy; (2) Preoperative receipt of NICT or NCT; (3) Clinical stage of locally advanced disease (cT1–4aN0–3M0), determined through comprehensive assessment including enhanced CT, upper gastrointestinal endoscopy ± endoscopic ultrasound, and neck/abdominal imaging, according to contemporary guidelines and the 8th edition AJCC/UICC TNM staging system for esophageal cancer; (4) Availability of complete clinical and pathological data. Exclusion criteria: (1) Presence of other primary malignant tumors; (2) Severe infection, autoimmune disease, or moderate to severe hepatic or renal insufficiency; (3) Loss to follow-up or missing key clinical or laboratory data.The detailed selection process is shown in Fig. 1.

### Data collection

Clinical and pathological data were collected from the hospital’s electronic medical record system, outpatient follow-up records, routine physical examination records, and annual telephone follow-ups. Baseline clinical characteristics collected included: age, sex, smoking history, drinking history, hypertension, diabetes, cardiovascular disease, height (cm), weight (kg), body mass index (BMI), clinical stage (cTNM), tumor location (upper/middle/lower thoracic esophagus or esophagogastric junction), tumor length (≤ 3 cm and > 3 cm), type of neoadjuvant therapy (NICT or NCT), number of treatment cycles, and treatment-related adverse events [graded according to Common Terminology Criteria for Adverse Events (CTCAE) version 5.0].

All laboratory indices were obtained from the most recent peripheral venous blood routine and biochemical tests performed within 1 week before surgery, after completion of neoadjuvant therapy. Laboratory indices collected included white blood cell count (WBC), neutrophil count (NEU), lymphocyte count (LYM), platelet count, serum albumin (ALB), and alanine aminotransferase (ALT).

NII was calculated exclusively from these routine preoperative blood and biochemical parameters obtained within 1 week before surgery after completion of neoadjuvant therapy, using the following formula: NII = ALB × (LYM/WBC) / (NEU/WBC) × ALT.

This composite index was designed to reflect the body’s nutritional status, lymphocyte/neutrophil balance, and liver function-related inflammatory status. To mitigate the influence of skewed distribution and extreme values, the natural logarithm (logNII = ln(NII)) of NII was taken, denoted as logNII, and used as a continuous variable in subsequent analyses; logNII was not centered or standardized.

Postoperative pathological slides were reviewed by at least two experienced pathologists. Pathological complete response (pCR) was defined as the absence of viable tumor cells in both the primary tumor site and all resected lymph nodes (ypT0N0) of the surgical specimen.

### Data analysis

All statistical analyses were performed using R software (version 4.3.1). After testing for normality, continuous data conforming to a normal distribution were presented as mean ± standard deviation and compared between groups using the independent samples t-test; data not conforming to a normal distribution were presented as median (interquartile range) and compared using the Mann-Whitney U test. Categorical data were presented as counts and percentages [n (%)] and compared between groups using the χ² test or Fisher’s exact test.

Using pCR as the dependent variable, univariate and multivariate logistic regression models were constructed. First, univariate logistic regression analysis was performed for each candidate clinical and laboratory variable to preliminarily screen covariates for inclusion in the multivariate model. Subsequently, in the multivariate logistic regression model, logNII was included as a continuous independent variable, and covariates were selected based on clinical relevance and univariate analysis results. Odds ratios (OR) and their 95% confidence intervals were estimated.

To characterize the potential nonlinear dose-response relationship between logNII and the probability of pCR, restricted cubic splines (RCS) were used to model logNII within the logistic regression framework. Models containing only logNII and models adjusted for covariates were fitted separately. Spline curves were plotted to visualize the relationship between logNII levels and the probability of achieving pCR. A likelihood ratio test was used to compare the model containing only the linear term of logNII with the model containing the RCS terms, to assess the contribution of the nonlinear component to the model fit. This informed the determination of the functional form of logNII in the multivariate model and its modeling approach as the primary predictor.

The discriminative ability of the predictive model was evaluated using the receiver operating characteristic (ROC) curve and the area under the curve (AUC). The clinical net benefit and potential application value of the model across different threshold probabilities were assessed using decision curve analysis (DCA). All tests were two-sided, and a P value < 0.05 was considered statistically significant.

## Results

### Comparison of baseline characteristics between pCR and non-pCR groups

This study included a total of 363 ESCC patients who underwent neoadjuvant therapy followed by radical esophagectomy. Among them, 100 patients (27.5%) achieved pCR (pCR group), while 263 patients (72.5%) did not (non-pCR group). No statistically significant differences were observed between the two groups regarding age distribution, comorbidities (hypertension, diabetes, cardiovascular disease), BMI grouping, tumor length, cT stage, cN stage, distribution of neoadjuvant treatment cycles, or baseline laboratory parameters including NEU and ALB (all *P* > 0.05), indicating overall comparability of baseline characteristics. Compared with the non-pCR group, patients in the pCR group had significantly higher logNII levels (0.28 ± 0.30 vs. 0.04 ± 0.31, *P* < 0.001), higher lymphocyte counts (1.91 ± 0.63 vs. 1.74 ± 0.60, *P* = 0.022), and lower ALT levels (15.69 ± 9.82 vs. 23.02 ± 21.13, *P* < 0.001). In addition, the pCR group had a higher proportion of females (45.0% vs. 24.0%, *P* < 0.001), and significantly greater proportions of patients without a smoking history (52.0% vs. 35.4%, *P* = 0.004) and without a drinking history (74.0% vs. 50.2%, *P* < 0.001). Regarding tumor location, the pCR group had a higher proportion of middle esophageal tumors (68.0% vs. 57.4%, *P* = 0.021). The proportion of patients receiving NICT was also significantly higher in the pCR group compared to the non-pCR group (86.0% vs. 72.2%, *P* = 0.006). See Table 1.

**Table 1:** Patient baseline characteristics

Variables	Total (*n* = 363)	Non-pcr (*n* = 263)	pcr (*n* = 100)	*P*
LogNII, Mean ± SD	0.10 ± 0.33	0.04 ± 0.31	0.28 ± 0.30	< 0.001
NEU, Mean ± SD	3.59 ± 1.85	3.69 ± 1.55	3.34 ± 2.46	0.106
LYM, Mean ± SD	1.79 ± 0.61	1.74 ± 0.60	1.91 ± 0.63	0.022
ALB, Mean ± SD	42.85 ± 3.93	42.73 ± 3.40	43.18 ± 5.08	0.325
ALT, Mean ± SD	21.00 ± 18.98	23.02 ± 21.13	15.69 ± 9.82	< 0.001
Gender, *n* (%)				< 0.001
Female	108 (29.75)	63 (23.95)	45 (45.00)	
Male	255 (70.25)	200 (76.05)	55 (55.00)	
Agegroup, *n* (%)				0.097
>65	149 (41.05)	101 (38.40)	48 (48.00)	
≤65	214 (58.95)	162 (61.60)	52 (52.00)	
Smoking history, *n* (%)				0.004
No	145 (39.94)	93 (35.36)	52 (52.00)	
Yes	218 (60.06)	170 (64.64)	48 (48.00)	
Drinking history, *n* (%)				< 0.001
No	206 (56.75)	132 (50.19)	74 (74.00)	
Yes	157 (43.25)	131 (49.81)	26 (26.00)	
Hypertension, *n* (%)				0.186
No	265 (73.00)	187 (71.10)	78 (78.00)	
Yes	98 (27.00)	76 (28.90)	22 (22.00)	
Diabetes, *n* (%)				0.301
No	332 (91.46)	243 (92.40)	89 (89.00)	
Yes	31 (8.54)	20 (7.60)	11 (11.00)	
Cardiovascular disease, *n* (%)				1.000
No	354 (97.52)	256 (97.34)	98 (98.00)	
Yes	9 (2.48)	7 (2.66)	2 (2.00)	
BMIgroup, *n* (%)				0.230
>24	131 (36.09)	90 (34.22)	41 (41.00)	
≤24	232 (63.91)	173 (65.78)	59 (59.00)	
Length, *n* (%)				0.772
>3	228 (62.81)	164 (62.36)	64 (64.00)	
≤3	135 (37.19)	99 (37.64)	36 (36.00)	
Tumor location, *n* (%)				0.021
Lower	111 (30.58)	91 (34.60)	20 (20.00)	
Middle	219 (60.33)	151 (57.41)	68 (68.00)	
Upper	33 (9.09)	21 (7.98)	12 (12.00)	
Ct Stage, *n* (%)				0.323
T1	4 (1.10)	2 (0.76)	2 (2.00)	
T2	26 (7.16)	19 (7.22)	7 (7.00)	
T3	321 (88.43)	231 (87.83)	90 (90.00)	
T4	12 (3.31)	11 (4.18)	1 (1.00)	
Cn Stage, *n* (%)				0.476
N0	84 (23.14)	57 (21.67)	27 (27.00)	
N1	109 (30.03)	83 (31.56)	26 (26.00)	
N2	133 (36.64)	94 (35.74)	39 (39.00)	
N3	37 (10.19)	29 (11.03)	8 (8.00)	
Treatment, *n* (%)				0.006
NCT	87 (23.97)	73 (27.76)	14 (14.00)	
NICT	276 (76.03)	190 (72.24)	86 (86.00)	
Cyclesgroup, *n* (%)				0.800
1	24 (6.61)	19 (7.22)	5 (5.00)	
2	254 (69.97)	185 (70.34)	69 (69.00)	
3	57 (15.70)	40 (15.21)	17 (17.00)	
>3	28 (7.71)	19 (7.22)	9 (9.00)	

Continuous variables are presented as mean ± standard deviation (SD); categorical variables are presented as number (%). Comparisons between non-pCR and pCR groups were performed using Student’s tt-test for continuous variables and the chi-square test (or Fisher’s exact test when expected frequencies were < 5) for categorical variables. LogNII: log-transformed Novel Inflammatory and Nutritional Index (not neutrophil-to-lymphocyte index)

*NEU* neutrophil count, *LYM* lymphocyte count, *ALB* serum albumin, *ALT* alanine aminotransferase, *BMI* body mass index, *NCT* neoadjuvant chemotherapy, *NICT* neoadjuvant immunochemotherapy

Bolded *P* values indicate statistically significant differences (*P* < 0.05)

### Nonlinear relationship between logNII and pCR

**Fig. 2:**
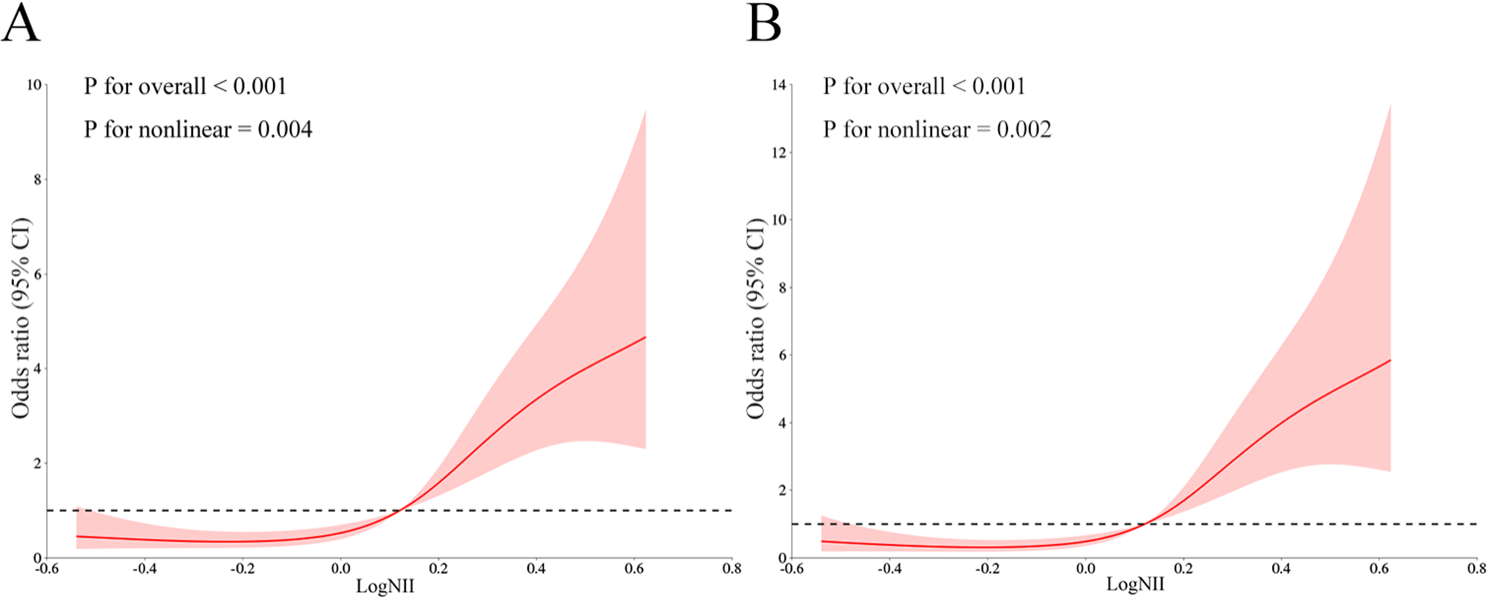
Association between logNII and odds of pCR. (**A)** Unadjusted analysis; (**B**) Multivariable-adjusted analysis. The solid line represents the odds ratio (OR) for pCR across the range of logNII values, with the shaded area indicating the 95% confidence interval (CI). The horizontal dashed line at OR = 1 represents the null effect. In both panels, the overall association was statistically significant (overall *P* < 0.001), and a nonlinear relationship was detected (nonlinearity *P* = 0.004 for (**A**) and P = 0.002 for (**B**)). Abbreviations: pCR, pathological complete response; logNII, log-transformed Nutrition-Inflammation Index

The dose-response relationship between logNII and pCR was assessed using restricted cubic splines within a logistic regression framework. In the unadjusted model, logNII showed a significant overall association with the probability of achieving pCR (overall *P* < 0.001), with a statistically significant nonlinear component (nonlinear *P* = 0.004), as shown in Fig. 2A. As logNII increased, the probability of pCR showed an overall upward trend, which became more pronounced at higher levels.

After further adjustment for covariates including sex, age, smoking history, drinking history, comorbidities, BMI, tumor length, tumor location, cT/cN stage, type of neoadjuvant therapy, and number of treatment cycles, the overall association between logNII and pCR remained significant in the multivariable-adjusted model (overall *P* < 0.001), and the nonlinear component retained statistical significance (nonlinear *P* = 0.002). The dose-response curve became steeper compared to the unadjusted model, suggesting that after controlling for potential confounders, higher levels of logNII were still associated with an increased probability of pCR, and this association exhibited a nonlinearly enhancing trend. See Fig. 2B.

### Confirmation of logNII as an independent correlate via multivariable model

In the multivariable logistic regression model with pCR as the outcome, covariates including sex, age group, smoking history, drinking history, hypertension, diabetes, cardiovascular disease, BMI group, tumor length, tumor location, cT stage, cN stage, type of neoadjuvant therapy, and number of treatment cycles were included, while logNII was modeled using restricted cubic splines.

Comparison between the full model containing the splined logNII and the reduced model without the logNII spline terms via the likelihood ratio test indicated a significant difference in model fit (χ² = 49.52, *P* < 0.001), suggesting that the inclusion of logNII significantly improved the model fit and that an independent and statistically significant association exists between logNII and pCR. Further comparison between the model containing only the linear term of logNII and the model containing the spline terms showed that the nonlinear component was at a significant level (*P* = 0.054), indicating that the relationship between logNII and pCR overall exhibited a monotonically increasing trend, while allowing for a certain degree of nonlinear curvature. Considering model robustness, the spline terms were retained in the final model.

In this multivariable model (see Table 2), besides logNII, several clinical variables were also independently associated with pCR: absence of drinking history (compared to presence; OR = 0.365, 95% CI: 0.173–0.752; note OR < 1 indicates drinking history is unfavorable for pCR), BMI > 24 kg/m² (compared to BMI ≤ 24 kg/m²; OR suggests an inverse relationship, see Table 2 for precise value), middle esophageal tumor location (compared to lower; OR = 2.053, 95% CI: 1.045–4.175), and receipt of NICT (compared to NCT; OR = 4.388, 95% CI: 2.066–9.963) were all associated with higher pCR rates.

**Table 2:** Multivariable logistic regression analysis for pCR

Variable	OR(95%CI)	*P*
logNII(spline, overall&nonlinearity)		NA
Intercept	0.436(0.006–21.756)	0.682
GenderMale	0.662(0.268–1.572)	0.357
Agegroup ≤ 65	0.738(0.418–1.300)	0.292
Smoking.historyYes	1.287(0.564–3.045)	0.555
Drinking.historyYes	0.365(0.173–0.752)	0.007
HypertensionYes	0.675(0.340–1.302)	0.249
DiabetesYes	1.086(0.410–2.781)	0.864
Cardiovascular.diseaseYes	0.760(0.088–4.738)	0.780
BMIgroup ≤ 24	0.509(0.276–0.929)	0.029
LengthLonger	0.997(0.563–1.774)	0.991
Tumor.locationMiddle	2.053(1.045–4.175)	0.041
Tumor.locationUpper	2.120(0.734–6.085)	0.161
cT.stageT2	0.762(0.048–10.641)	0.838
cT.stageT3	0.828(0.063–9.487)	0.877
cT.stageT4	0.169(0.003–4.724)	0.319
cN.stageN1	0.620(0.283–1.353)	0.230
cN.stageN2	0.785(0.378–1.634)	0.515
cN.stageN3	0.732(0.234–2.152)	0.579
TreatmentNICT	4.388(2.066–9.963)	< 0.001
Cyclesgroup1	0.658(0.140–2.914)	0.585
Cyclesgroup2	0.876(0.316–2.601)	0.804
Cyclesgroup3	1.455(0.432–5.149)	0.551

*OR* odds ratio, *CI* confidence interval, *Ref*. reference category

LogNII was modeled with restricted cubic splines; its overall and nonlinear association is shown separately in Table 3. The model shows that TreatmentNICT (neoadjuvant immunochemotherapy) was strongly associated with pCR (OR=4.388, 95% CI:2.066–9.963, *P* < 0.001). Drinking history, BMI ≤ 24, and middle tumor location were also significant predictors (*P* < 0.05)

**Fig. 3:**
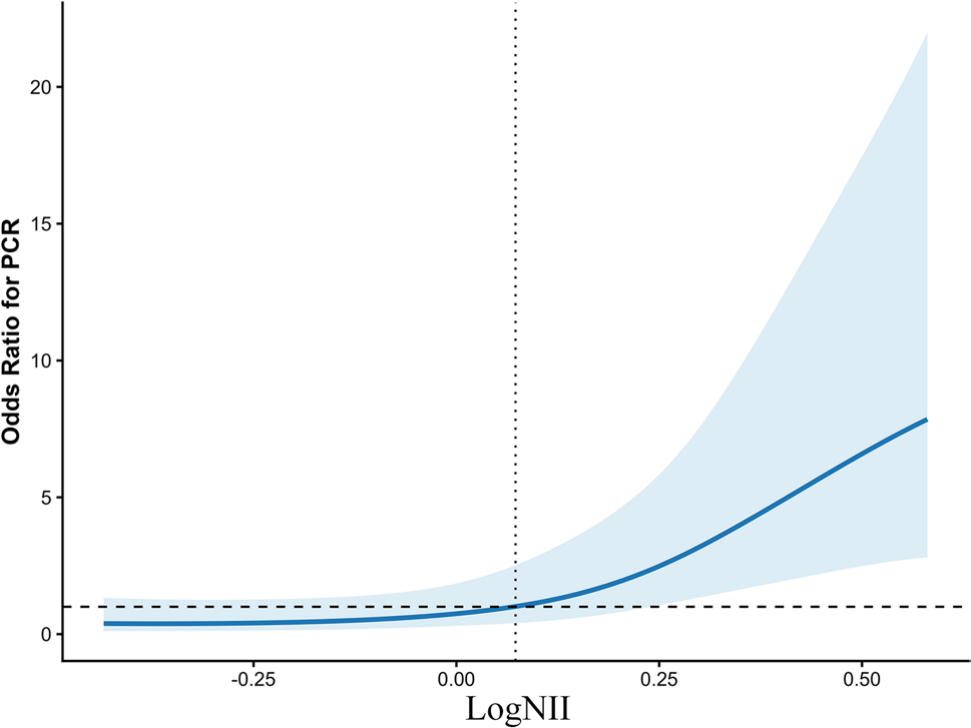
Visual representation of the odds ratio trend for pCR across logNII values. This figure provides a simplified graphical summary of the relationship between logNII and the likelihood of achieving pCR, illustrating the general trend of increasing odds with higher logNII levels

To more intuitively quantify the effect of different levels of logNII, we calculated the adjusted ORs for different percentiles of logNII relative to its median level based on the spline model (see Table 3). Holding other covariates constant, compared to the median logNII level (0.07), the ORs for achieving pCR at the P75 (0.325) and P90 (0.479) levels were 3.59 (95% CI: 1.49–8.64) and 6.22 (95% CI: 2.37–16.32), respectively, indicating that high logNII levels are associated with significantly increased probability of pCR. In contrast, the ORs for the lower percentiles (P10, P25) were below 1, but the confidence intervals were wide, suggesting less precise estimates in the low-level range. Synthesizing these results, logNII remains an independent predictor of pCR after multivariable adjustment, and its effect overall exhibits a monotonically increasing nonlinear trend. See Fig. 3; Table 2, and Table 3.

**Table 3:** Adjusted ORs at different percentiles of logNII relative to the median

Point	logNII	OR	CI_low	CI_high
10%	-0.333	0.383	0.117	1.259
25%	-0.18	0.45	0.155	1.31
50%	0.07	1	0.402	2.489
75%	0.325	3.592	1.492	8.644
90%	0.479	6.224	2.373	16.322

ORs are expressed relative to the median value of logNII (50th percentile, OR = 1). Higher logNII levels are associated with increased odds of pCR, reaching statistical significance at the 75th and 90th percentiles

### Model performance validation

**Fig. 4:**
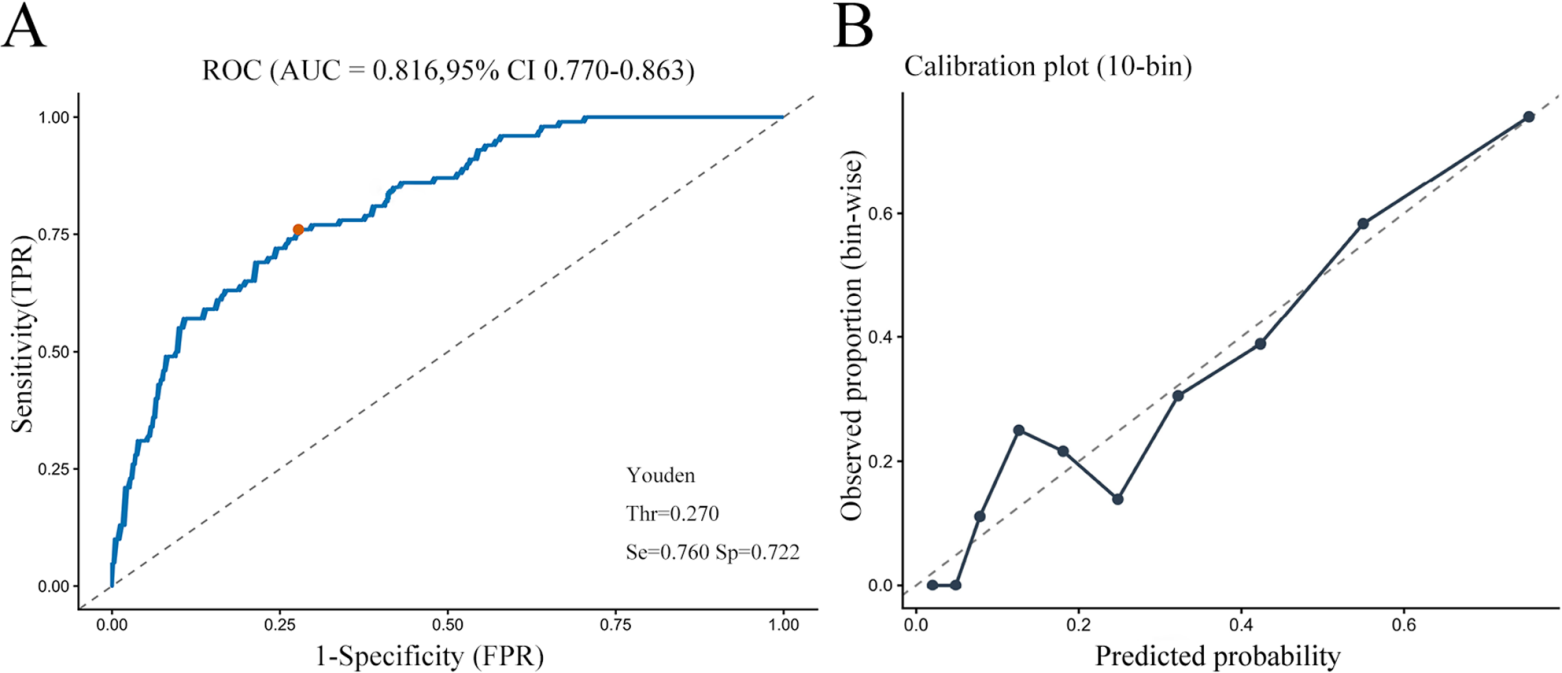
Model performance. (**A) **ROC curve (AUC = 0.816, 95% CI: 0.770–0.863; optimal cut-off = 0.270, sensitivity = 0.760, specificity = 0.722). (**B)** Calibration curve showing predicted vs. observed pCR probabilities across 10 bins; diagonal line indicates perfect calibration

The multivariable model constructed based on logNII and clinical covariates demonstrated good discriminative ability in distinguishing between pCR and non-pCR patients. ROC curve analysis showed that the model’s AUC for predicting pCR was 0.816 (95% CI: 0.770–0.863), as shown in Fig. 4A; Table 4. The optimal cutoff value determined by the Youden Index was 0.27, at which the model’s sensitivity was 76.0%, specificity was 72.2%, positive predictive value was 51.0%, and negative predictive value was 88.8%. This indicates that at this threshold, the model achieved a good balance between sensitivity and specificity, exhibiting high accuracy particularly in ruling out patients unlikely to achieve pCR.

**Table 4:** Performance metrics of the predictive model from ROC curve analysis

AUC	AUC_CI_L	AUC_CI_U	Thr_Youden	Sens_at_Thr	Spec_at_Thr	PPV_at_Thr	NPV_at_Thr
0.816	0.77	0.863	0.27	0.76	0.722	0.51	0.888

*AUC* area under the ROC curve, *CI* confidence interval, *Sens* sensitivity, *Spec* specificity, *PPV* positive predictive value, *NPV* negative predictive value, *Thr_Youden* threshold corresponding to the maximum Youden’s index. The model demonstrates good discriminative ability with an AUC of 0.816 (95% CI:0.770–0.863)

Calibration assessment showed acceptable agreement between predicted and observed probabilities of pCR. In the 10-bin calibration plot, the observed proportions generally followed the 45° reference line, particularly in the intermediate-to-high probability range, although some deviation was observed at lower predicted probabilities. See Fig. 4B.

**Fig. 5:**
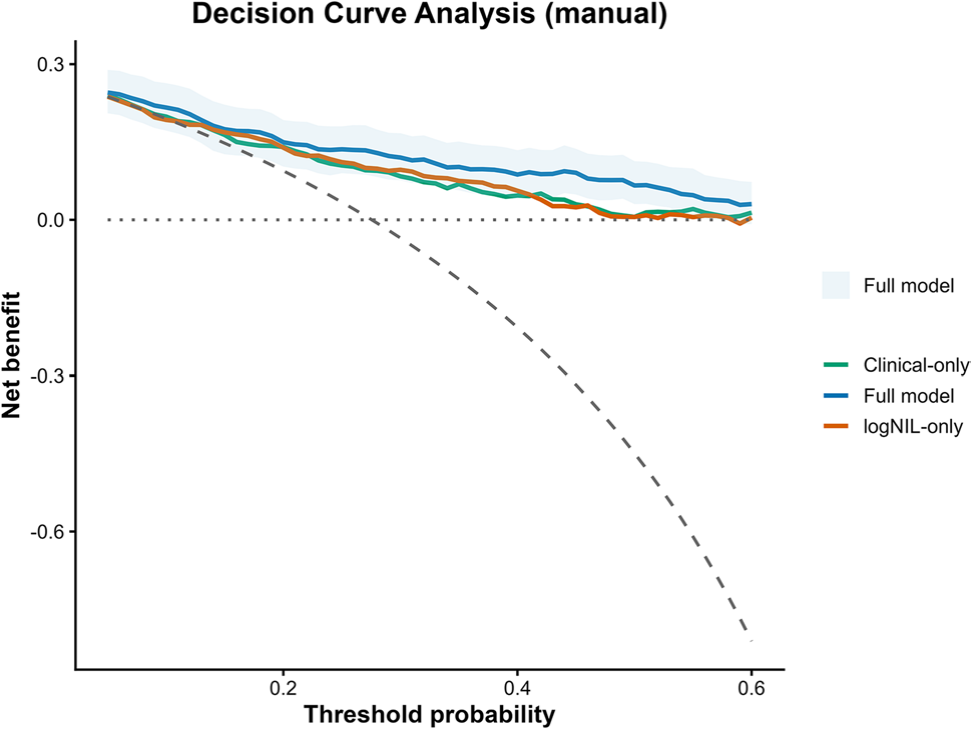
Decision curve analysis for the prediction of pCR. The net benefit of using the prediction models is plotted against the threshold probability. The curves represent the full model (incorporating logNII and clinical variables), the clinical-only model, and the logNII-only model. The “treat all” and “treat none” strategies are shown as reference lines. The full model demonstrates the highest net benefit across a clinically relevant range of threshold probabilities, supporting its clinical utility

Decision curve analysis results showed that across the threshold probability range of approximately 0.05–0.57, the “Full Model” (clinical variables + logNII) consistently yielded a positive net benefit with a 95% confidence interval not crossing zero, and overall performed better than both the “Clinical Variables Only Model” and the “logNII Only Model”, see Fig. 5. Near the threshold probability of 0.05, the maximum net benefit (NB) of the Full Model was approximately 0.245; compared to the Clinical Only Model, the maximum incremental net benefit (ΔNB = 0.068) occurred around a threshold probability of 0.49. These results suggest that across a broad range of clinical decision thresholds, incorporating logNII provides added value for clinical decision-making based on pCR risk prediction.

## Discussion

This study systematically integrated clinicopathological characteristics with existing clinically available markers to thoroughly investigate the predictive value of NII for treatment response to neoadjuvant therapy in esophageal squamous cell carcinoma. To our knowledge, this study employs restricted cubic spline analysis, precisely revealing an independent nonlinear dose-response relationship between logNII and pathological complete response (pCR), a finding that may address the limitations of traditional linear models. The prediction model established based on this demonstrates outstanding discriminative ability (AUC = 0.816) and good calibration, It demonstrated superior performance compared to various previously reported predictive models [11, 12]. More importantly, decision curve analysis confirmed that the model provides significant net clinical benefit across a wide range of threshold probabilities. These research results may provide a potentially useful quantitative tool for individualized treatment decision-making in ESCC patients and may offer a new perspective for host immune and inflammatory milieu research, although this warrants external validation [13].

As the gold standard for evaluating neoadjuvant therapy efficacy, the clinical value of pCR has been fully validated in multiple large-scale clinical studies [3, 18]. Research indicates that the 5-year survival rate of ESCC patients achieving pCR can exceed 60%, significantly better than non-pCR patients [5]. This marked survival difference makes accurate pCR prediction a key link in optimizing treatment strategies.

It is important to clarify that NII is a composite inflammatory-nutritional index derived exclusively from routine preoperative blood and biochemical parameters, rather than a direct histological measure of the tumor microenvironment. As such, NII indirectly reflects the host’s systemic inflammatory, immune, nutritional, and metabolic status, and does not directly quantify local tumor immune infiltration.Its association with pCR, although robust in our analysis, is indirect and should not be interpreted as a direct quantification of local tumor immune infiltration. For example, the ALT component of NII is traditionally linked to hepatic function; our interpretation is that ALT may reflect hepatic and metabolic status relevant to systemic inflammatory balance and treatment tolerance, rather than directly indicating the intensity of anti-tumor immune responses. The precise biological mechanisms linking NII to pCR remain to be further investigated in future translational studies.

Beyond logNII, this study revealed several other clinically significant predictive factors. Neoadjuvant immunochemotherapy (NICT) showed the strongest predictive efficacy (OR = 4.388), a result consistent with the current important status of immunotherapy in the field of esophageal cancer [6] and providing new evidence for the advantages of immune combination therapy [15]. Furthermore, middle tumor location was confirmed to be associated with a higher pCR rate, which might be related to the specific anatomical characteristics and lymphatic drainage patterns of this location [7]. By constructing a nomogram model incorporating multiple independent predictive factors, we achieved precise quantitative prediction of pCR probability for individual patients. This model demonstrated stability in both the training cohort and Bootstrap validation, and decision curve analysis proved its practical application value in clinical decision-making [19].

It must be noted that, as a single-center retrospective study, this research inevitably carries a risk of selection bias [20]. Although we employed the Bootstrap method for sufficient internal validation, the model’s external validity still requires further verification through prospective, multi-center studies [21]. Furthermore, NII is a composite laboratory index that does not provide information on specific immune cell subsets or molecular features of the tumor microenvironment. Future studies incorporating techniques such as multiplex immunofluorescence or flow cytometry may allow deeper analysis of the roles of specific immune cell populations like CD8 + T cells and Tregs [17], which could complement the predictive information provided by NII. Furthermore, SCC antigen was not routinely available in our center and had substantial missingness; therefore it could not be reliably incorporated into the comparative table.Additionally, this study did not include some emerging biomarkers such as PD-L1 expression level and tumor mutational burden [22–24], which may offer complementary predictive information. Nevertheless, these limitations do not affect the reliability of the main conclusions of this study and instead point the way for future research.

## Conclusions

In summary, this study established NII as a strong and independent predictor of pCR following neoadjuvant therapy in ESCC, demonstrating a nonlinear relationship. We developed and validated a nomogram prediction model that integrates NII with clinical factors, thus may serve as a potentially useful tool for constructing individualized neoadjuvant treatment strategies.However, external validation in prospective multicenter cohorts is warranted before clinical implementation.

## Data Availability

The datasets used and/or analysed during the current study are available from the corresponding author on reasonable request.
